# From metabolic byproduct to immune modulator: the role of lactate in tumor immune escape

**DOI:** 10.3389/fimmu.2024.1492050

**Published:** 2024-11-25

**Authors:** Mengqian Jiang, Yuanchun Wang, Xiaoyong Zhao, Jinming Yu

**Affiliations:** ^1^ School of Clinical Medicine, Shandong Second Medical University, Weifang, China; ^2^ Department of Radiation Oncology and Shandong Provincial Key Laboratory of Precision Oncology, Shandong Cancer Hospital and Institute, Shandong First Medical University and Shandong Academy of Medical Sciences, Jinan, Shandong, China; ^3^ Laboratory of Anesthesia and Critical Care Medicine in Colleges and Universities of Shandong Province, School of Anesthesiology, Shandong Second Medical University, Weifang, China; ^4^ Department of Anesthesiology, Shandong Cancer Hospital and Institute, Shandong First Medical University and Shandong Academy of Medical Sciences, Jinan, Shandong, China; ^5^ Department of Radiation Oncology and Shandong Provincial Key Laboratory of Precision Oncology, Shandong First Medical University and Shandong Academy of Medical Sciences, Jinan, Shandong, China; ^6^ Research Unit of Radiation Oncology, Chinese Academy of Medical Sciences, Jinan, Shandong, China

**Keywords:** lactic acid, immune evasion, tumor microenvironment, combination therapy, tumor immune

## Abstract

Lactic acid, a key metabolic byproduct within the tumor microenvironment, has garnered significant attention for its role in immune evasion mechanisms. Tumor cells produce and release large amounts of lactic acid into the tumor microenvironment through aberrant glycolysis via the Warburg effect, leading to a drop in pH. Elevated lactic acid levels profoundly suppress proliferation capacity, cytotoxic functions, and migratory abilities of immune effector cells such as macrophages and natural killer cells at the tumor site. Moreover, lactic acid can modulate the expression of surface molecules on immune cells, interfering with their recognition and attack of tumor cells, and it regulates signaling pathways that promote the expansion and enhanced function of immunosuppressive cells like regulatory T cells, thereby fostering immune tolerance within the tumor microenvironment. Current research is actively exploring strategies targeting lactic acid metabolism to ameliorate tumor immune evasion. Key approaches under investigation include inhibiting the activity of critical enzymes in lactic acid production to reduce its synthesis or blocking lactate transporters to alter intracellular and extracellular lactate distribution. These methods hold promise when combined with existing immunotherapies such as immune checkpoint inhibitors and chimeric antigen receptor T-cell therapies to enhance the immune system’s ability to eliminate tumor cells. This could pave the way for novel combinatorial treatment strategies in clinical cancer therapy, effectively overcoming tumor immune evasion phenomena, and ultimately improving overall treatment efficacy.

## 1 Background

Lactic acid is a key metabolic product resulting from anaerobic glucose metabolism. In hypoxic environments, cells primarily generate energy through anaerobic respiration, accompanied by the conversion of pyruvate into lactic acid (1). During intense exercise, when oxygen supply is insufficient, muscle cells rapidly undergo anaerobic glycolysis, producing a large amount of lactic acid. The accumulation of lactic acid leads to a localized decrease in pH, resulting in the sensation of soreness or acidity. However, lactic acid is not merely a metabolic waste product; it can also be transported via the bloodstream to the liver, where it can be converted back into glucose through gluconeogenesis, serving as an important supplementary source of energy within the body (2). Within the human gastrointestinal tract, certain probiotics can also produce lactic acid, which helps maintain the stability of the gut environment and inhibits the growth of harmful bacteria. However, when severe tissue hypoxia occurs, such as during shock, heart failure, or pulmonary diseases, excessive production of lactic acid by cells surpasses the body’s clearance capacity, leading to elevated blood lactate levels and potentially causing lactic acidosis, which in turn impacts normal physiological functions of the organism (3). Interestingly, tumor cells exhibit a preference for glycolysis to produce large amounts of lactic acid even under aerobic conditions, a phenomenon known as the Warburg effect (4). The metabolic reprogramming of tumor cells involves multiple mechanisms: Firstly, they utilize growth factor signaling to drive abnormal proliferation. Secondly, the activation of the PI3K-AKT signaling pathway significantly influences cell survival status and metabolic rates. Concurrently, in order to meet their rapidly growing energy demands and biosynthetic requirements, tumor cells increase the number of glucose transporters, thereby taking up more glucose and further enhancing glycolysis, even preferring this energy-producing pathway under aerobic conditions (5). Tumor cell metabolic reprogramming is a complex process involving multi-level and multi-factor interactions. This process encompasses everything from the activation of key transcription factors such as HIF-1α, c-Myc, and p53, to epigenetic modifications like DNA methylation and histone acetylation, all of which finely regulate the expression of metabolic genes. Tumor cells exhibit a high degree of metabolic plasticity and adaptability to meet their biosynthetic needs and respond to changes in the microenvironment by enhancing glycolysis, regulating oxidative phosphorylation, activating fatty acid synthesis, and glutamine metabolism (6). In the tumor microenvironment, metabolites such as lactate not only shape the metabolic state of tumor cells but also play a key role in tumor immune evasion by regulating the function of immune cells, such as suppressing T cell activity and promoting the accumulation of immunosuppressive cells (7). Additionally, metabolic reprogramming is closely related to the invasiveness, metastatic ability, and therapeutic resistance of tumors, with metabolic intermediates potentially acting as signaling molecules involved in the regulation of the tumor microenvironment (8). Targeted therapeutic strategies aimed at tumor cell metabolic pathways, such as by inhibiting specific metabolic enzymes or modulating the activity of metabolic pathways, provide new opportunities for cancer treatment. However, the complexity and plasticity of metabolic pathways also present challenges, including treatment specificity, the development of drug resistance, and off-target effects. Therefore, future treatment strategies need to be tailored based on the metabolic characteristics of individual tumors, achieving precision treatment through metabolic typing and the identification of biomarkers (9). Moreover, the regulation of metabolic reprogramming may enhance or inhibit the effects of immunotherapy. Modulating the levels of metabolites in the tumor microenvironment may enhance the infiltration and activity of immune cells, thereby improving the efficacy of immune checkpoint inhibitors (10). Therefore, delving into the complex network of tumor cell metabolic reprogramming is crucial for developing more effective cancer treatment strategies. This may involve the combined application of metabolic targeted therapy and immunotherapy to overcome tumor immune evasion and improve treatment outcomes, bringing more effective treatment options to patients. Tumor-associated macrophages (TAMs) exhibit functional plasticity across different stages of tumor development. TAMs accumulate in hypoxic regions and express HIF-1α, where they regulate glycolytic genes to preferentially utilize glycolysis as a means to maintain their inflammatory phenotype, thereby promoting tumor-related inflammation. Concurrently, the glycolytic product lactate exerts a significant influence on TAM polarization, driving them towards a protumor phenotype, as well as indirectly facilitating tumor progression and immune evasion through its effects on the functionality of immune cells (5). Under normal circumstances, the immune response can effectively identify and eliminate tumor cells; however, this mechanism often fails in cancer patients, and the reasons for the inability to clear tumors are not yet fully understood. It is currently believed that this inefficiency is closely related to immune evasion by tumor cells. Immune evasion refers to the process by which pathogens or tumor cells use various mechanisms to avoid recognition and attack by the host’s immune system. Research has shown that lactic acid plays an indirect but significant role in modulating immune responses. For instance, within the tumor microenvironment, high concentrations of lactic acid can lower the local pH, thereby suppressing the anti-tumor activities of macrophages and NK cells, thus facilitating immune escape by tumor cells (5). Lactic acid may also influence the activation and function of immune cells through pathways such as the AMPK signaling pathway and TGF-β signaling pathway, thereby promoting tumor cells’ evasion from immune system clearance (11). Recent research developments have revealed the multifaceted role of L-lactate in tumor immune evasion. Specific sensors AARS1 and AARS2 can sensitively detect changes in intracellular L-lactate concentrations and trigger a series of L-lactate-dependent ATP-activated lactylation reactions. This process leads to lactylation modification of the cGAS enzyme, shifting its interaction with DNA from an active state to an inhibitory state, thereby finely regulating the cell’s immune response (12). In gastric cancer research, lactate, through its interaction with the receptor GPR81, upregulates the expression of CX3CL1, promoting the migration of regulatory T cells (Tregs) to the tumor microenvironment, thereby enhancing the tumor’s immune resistance (13). This finding highlights the important role of lactate in shaping the tumor microenvironment and promoting immune evasion. In prostate cancer research, lactate released by cancer-associated fibroblasts (CAFs) has a significant impact on the polarization process of CD4+ T cells. Lactate secreted by CAFs not only reduces the Th1 cell subset with antitumor activity but also promotes the increase of Tregs through lactate-dependent SIRT1-mediated deacetylation of transcription factors, activation of NF-kB, and expression of FoxP3, thereby inhibiting effective antitumor immune responses (14). Dichloroacetate (DCA), as a potential immunotherapy drug, directly acts on myeloid cells by inhibiting the activity of pyruvate dehydrogenase kinases (PDKs), effectively suppressing the immunosuppressive effects caused by lactate. DCA can reduce the expression of arginase I (Arg1) in macrophages and promote the proliferation of CD8+ T cells, thus potentially serving as a new immunotherapy strategy to enhance the body’s antitumor immune response (15). In summary, these findings not only deepen our understanding of the role of lactate in tumor immune evasion but also provide a scientific basis for the development of new immunotherapy strategies. By targeting lactate metabolism, we hope to overcome tumor-induced immunosuppression and thereby improve the efficacy of cancer treatment. Thus, while lactic acid itself is not a direct mechanism of immune evasion, under pathological conditions, it may contribute to the creation of an environment unfavorable for the effective functioning of the immune system by influencing cellular metabolism and remodeling the microenvironment, thereby promoting the occurrence of tumor immune evasion.

## 2 Lactic acid metabolism

### 2.1 The principles of lactic acid metabolism

**Figure 1:**
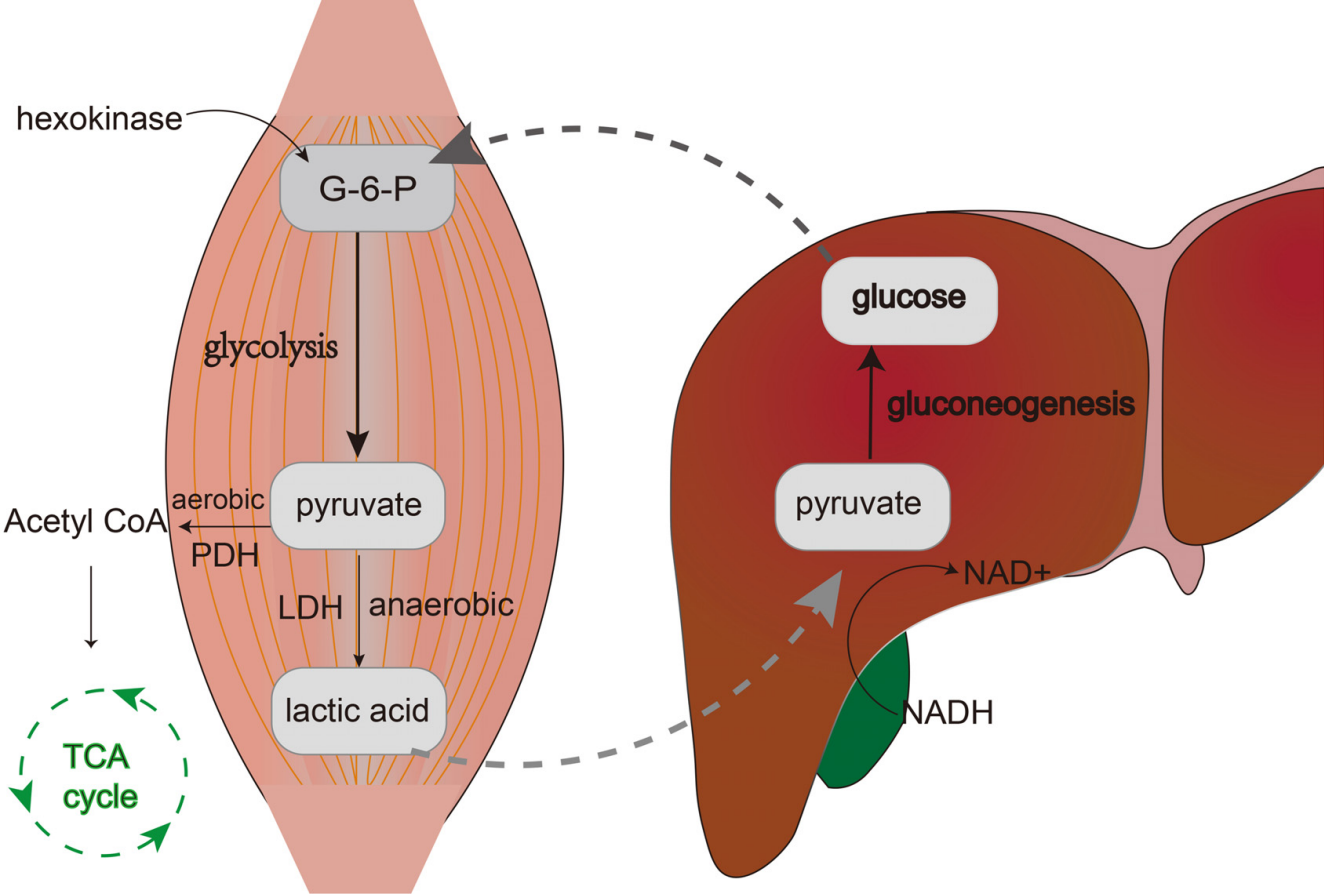
The figure shows the process of the lactic acid cycle. Within muscle cells, glucose is converted to glucose-6-phosphate (G-6-P) by hexokinase, which then undergoes glycolysis and breaks down into pyruvate. Under anaerobic conditions, pyruvate is catalyzed by lactate dehydrogenase (LDH) to form lactate. Lactate is then transported via the bloodstream to the liver where it is reconverted into glucose through gluconeogenesis.

Glucose is initially converted into glucose-6-phosphate within the cell under the catalysis of hexokinase. This glucose-6-phosphate then undergoes transformation into fructose-1,6-bisphosphate by the action of phosphofructokinase-1. Ultimately, fructose-1,6-bisphosphate is converted to pyruvate with the aid of pyruvate kinase. Under aerobic conditions, pyruvate is further metabolized through the action of pyruvate dehydrogenase, yielding acetyl-CoA, which enters the Tricarboxylic Acid Cycle (TCA cycle), also known as the Krebs cycle, leading to the production of a substantial amount of ATP (adenosine triphosphate).In anaerobic conditions, however, pyruvate is converted into lactate via the catalysis of lactate dehydrogenase (LDH), releasing a small amount of energy in the process (16, 17). During high-intensity exercise, when oxygen supply is insufficient, large amounts of lactate are produced within muscle cells. However, the lactate generated in muscles does not accumulate for long periods; instead, it undergoes a series of transport, conversion, release, and recycling processes that form a closed metabolic loop. Lactate exits muscle cells with the aid of monocarboxylate transporters (MCTs) and enters the bloodstream, where it circulates to the liver. In the liver, lactate experiences gluconeogenesis, being reconverted back into pyruvate, which ultimately leads to the synthesis of glucose (2). The newly synthesized glucose is released into the bloodstream, becoming a part of the blood glucose that can be utilized by various tissues throughout the body, particularly muscle cells (2). Thus, the lactate produced by muscles under anaerobic conditions is recycled and reused in a closed metabolic loop known as the Lactic Acid Cycle, or Cori cycle. The significance of this cycle lies in the fact that cells are not only able to continue generating energy under oxygen-deprived conditions but also prevent the accumulation of excessive lactate within the cells, which could otherwise lead to acidosis (18). Moreover, it plays a crucial role in maintaining glucose homeostasis and energy distribution (**Figure 1**).

The understanding of the lactate cycle has seen significant updates in recent years, leading to a revised model of the lactate cycle. This new model not only considers the role of lactate as an energy substrate but also emphasizes the various functions of lactate in the tumor microenvironment (19). Lactate exerts immunosuppressive functions in the tumor microenvironment through multiple mechanisms. Studies have shown that lactate can inhibit the activity of T cells and promote the accumulation of immunosuppressive cells, such as regulatory T cells and myeloid-derived suppressor cells (MDSCs) (11, 20, 21). For instance, lactate inhibits the proliferation and cytokine production of dendritic cells and T cells by affecting their function (11, 20, 21). Additionally, lactate can promote the M2-like polarization of tumor-associated macrophages, further suppressing the immune response (11). The accumulation of lactate can lower the pH value of the tumor microenvironment, which is conducive to the invasion and metastasis of tumor cells (22, 23). The low pH environment is not only beneficial for the survival and spread of tumor cells but also affects the efficiency of drug delivery and the function of immune cells (22, 23). The lactate cycle involves the exchange of metabolites between tumor cells and surrounding stromal cells, which helps the growth and survival of tumors (23, 24). Lactate, as an energy-rich metabolite, serves as a precursor for gluconeogenesis and ATP synthesis, playing an important role in the metabolic shuttling within the tumor microenvironment (23, 24). Moreover, tumor cells can adjust their metabolic pathways in response to changes in the microenvironment, including switching the production and utilization of lactate under hypoxic and aerobic conditions (23, 24). The plasticity of lactate metabolism provides tumor cells with the ability to adapt to different microenvironmental conditions. The revised lactate cycle model emphasizes the complex role of lactate in tumor biology, especially its role in immune modulation, pH regulation, energy and metabolite exchange, and metabolic plasticity. These findings provide an important theoretical basis for the development of new cancer treatment strategies.

### 2.2 The functions of lactic acid in normal physiological conditions

Under conditions of adequate oxygen supply, lactic acid is not a primary energy source. However, when cells are under hypoxic or anaerobic conditions, glycolysis is accelerated, and the resulting lactic acid serves as an emergency energy source. Lactate can be regenerated into glucose through gluconeogenesis and subsequently yield more ATP via aerobic metabolism (25). When lactic acid accumulates within cells, intracellular acidification occurs, which can lead to local metabolic acidosis. However, lactic acid can be transported via the bloodstream to the liver and other organs for further metabolism, thereby contributing to the restoration of acid-base balance within the body (26). Under normal physiological conditions, lactate produced by muscles is transported to the liver through the Cori cycle, where it undergoes gluconeogenesis to be converted back into glucose and released into the bloodstream, providing energy for tissues throughout the body, especially energy-demanding ones like the brain and muscles (27). This cycle not only contributes to the redistribution of energy but also plays a critical role in maintaining blood sugar stability (28). Research has also revealed that lactate, through its signaling functions, regulates cellular communication and metabolism, and participates in the regulation of biological processes such as inflammatory responses, cell proliferation, and differentiation (29). As a signaling molecule, lactate plays a significant role in regulating energy metabolism, tumor growth and metastasis, immune escape mechanisms, energy supply of neuronal cells, synaptic plasticity, learning and memory processes, and cell proliferation and differentiation through carriers and receptors such as MCTs and GPR81. The transport and utilization of lactate in tumor cells through monocarboxylate transporters such as MCT1 and MCT4 are crucial for the energy supply of tumor cells. Studies have shown that MCT1 is one of the main pathways for lactate uptake, especially in human cervical cancer cells that utilize lactate for oxidative metabolism (30, 31). Additionally, MCT4 drives lactate release under hypoxic conditions, while MCT1 is responsible for taking up lactate into tumor cells and tumor endothelial cells (30, 31). These findings reveal the important role of lactate in the tumor microenvironment, including promoting tumor angiogenesis and growth (32). GPR81 is expressed in various cancer cell lines, and its expression level is correlated with the speed of pancreatic cancer tumor growth and metastasis (32). Furthermore, the activation of GPR81 can suppress the expression of PD-L1, which may promote immune surveillance (33). Lactate is also involved in regulating the energy supply of neuronal cells and synaptic plasticity. Studies have shown that lactate can affect the function of Müller cells, including glucose metabolism and mitochondrial function, by activating the GPR81 receptor (34). Additionally, lactate can regulate the dialogue between neurons and astrocytes, providing an energy substrate for neurons through the activation of the GPR81 receptor (35). In the tumor microenvironment, the accumulation of lactate leads to a decrease in local pH, which not only promotes the invasiveness and metastasis of tumor cells but also affects drug distribution and the efficacy of immune cells (36, 37). The accumulation of lactate in the TME not only affects the behavior of tumor cells by directly changing the local pH but also indirectly affects tumor development and treatment response by regulating the function of immune cells and drug distribution. In the field of inflammatory responses, the role of lactate is also significant. The role of lactate in inflammatory responses is multifaceted, and its function in inflammatory diseases is mainly reflected in the regulation of macrophage polarization. In an inflammatory environment, the accumulation of lactate can regulate the function of macrophages, prompting them to polarize towards the M2 type. Lactate exerts its effects through specific receptors such as GPR81, inhibiting the activity of the NLRP3 inflammasome, reducing the production of inflammatory mediators, and thus playing an anti-inflammatory role (38). This anti-inflammatory effect helps prevent excessive inflammatory responses, thereby maintaining tissue homeostasis (39). However, the role of lactate in the tumor microenvironment is also dualistic. On the one hand, lactate can play an anti-inflammatory role by regulating the function of macrophages and inhibiting the activity of the inflammasome; on the other hand, it promotes the development of tumors by suppressing antitumor immune responses in the tumor microenvironment (40, 41). This dual role makes lactate a potential target for the treatment of inflammatory diseases and tumors (42). (**Table 1**) In summary, under normal physiological conditions, lactate plays a significant role in energy homeostasis and contributes to maintaining overall metabolic stability through multiple mechanisms.

**Table 1:** The role and mechanisms of lactate in different biological processes.

Biological Process	Mechanism of Lactic Acid	Related Receptors	Key Findings	References
Energy Metabolism	Acts as an energy substrate and influences cellular energy status	MCT1, MCT4	MCT1 is the primary pathway for lactate uptake; MCT4 drives lactate release under hypoxic conditions.	(29, 30)
Tumor Growth and Metastasis	Through transport by MCTs within tumor cells providing energy; promotes angiogenesis and tumor growth	MCT1, MCT4, GPR81	Accumulation of lactate in the tumor microenvironment leads to a decrease in pH, promoting invasiveness and metastasis; expression of GPR81 correlates with the rate of tumor growth and metastasis.	(31)
Immune Evasion Mechanism	Inhibits PD-L1 expression, potentially promoting immune surveillance	GPR81	Activation of GPR81 inhibits PD-L1 expression, suggesting its role in regulating tumor immune evasion.	(32)
Energy Supply in Neural Cells	Influences Müller cell function through activation of the GPR81 receptor	GPR81	Impacts glucose metabolism and mitochondrial function; regulates dialogue between neurons and astrocytes.	(33, 34)
Synaptic Plasticity	Influences synaptic plasticity through activation of the GPR81 receptor	GPR81	Provides energy substrates for neurons.	(33, 34)
Learning and Memory	Influences dialogue between neurons and astrocytes through the GPR81 receptor	GPR81	Provides energy substrates for neurons.	(33, 34)
Inflammatory Response	Regulates macrophage polarization towards M2 phenotype; inhibits NLRP3 inflammasome activity	GPR81	Reduces inflammatory mediator production, exerting anti-inflammatory effects; helps maintain tissue homeostasis.	(37, 38)
Tumor Microenvironment	Plays an anti-inflammatory role by modulating macrophage function and inhibiting inflammasome activity; promotes tumor progression by suppressing antitumor immune responses	MCTs, GPR81	Dual roles make lactate a potential therapeutic target for inflammatory diseases and tumors.	(35, 36, 39, 40)

### 2.3 The impact of lactic acid accumulation under abnormal conditions

Under hypoxic or anoxic conditions, such as during intense exercise, shock, or pneumonia, cells primarily rely on anaerobic glycolysis for energy production, which concurrently results in the generation of a large amount of lactic acid that is released into the cytoplasm (43). Lactic acid accumulation is a dynamic process involving both production and clearance aspects. Under normal circumstances, lactic acid in the blood is transported to the liver where it is eventually converted back into glucose through gluconeogenesis. Additionally, a portion of lactic acid can be taken up by muscles and other tissues during aerobic recovery periods, and within these tissues’ mitochondria, it undergoes complete oxidation to carbon dioxide and water via the tricarboxylic acid (TCA) cycle, also known as the Krebs cycle (44). However, in pathological states such as septic shock or liver failure, due to inadequate blood perfusion or decreased hepatic metabolic capacity, the mechanisms for clearing lactic acid are impaired. As a result, lactic acid cannot be promptly converted and eliminated from the body, leading to elevated levels of lactate in the blood, a condition known as hyperlactatemia. Lactic acidosis not only disrupts acid-base balance, causing a decrease in blood pH, but also affects the stability of the intracellular environment, inhibits enzyme activity, interferes with energy metabolism, further exacerbating cellular dysfunction, and can even culminate in multi-organ failure (45). Moreover, local accumulation of lactic acid in tissues can lead to tissue edema, heightened inflammatory responses, and increased microvascular permeability, thereby exacerbating tissue injury. In the context of immune regulation, lactate functions as a significant metabolic signaling molecule, and its concentration changes directly influence the function of immune cells. For instance, lactate can modulate the activity of key transcription factors, impacting the activation and differentiation of immune cells, thus influencing immune responses. Additionally, the local concentration of lactic acid within tissues can guide the migration of immune cells to specific areas because certain immune cells express receptors on their surface that sense lactate concentration gradients, guiding their chemotactic movement (46). In summary, lactate plays a pivotal role in a variety of diseases, functioning both as a significant constituent of energy metabolism and as a signaling molecule participating in diverse pathophysiological processes. Notably, it exerts a considerable influence within immune regulation, affecting the functionality of immune cells and thereby either promoting or dampening inflammatory responses. This multifaceted action contributes to immune evasion in conditions such as tumors and inflammatory diseases. Moreover, lactate serves as a biomarker in many diseases, aiding in diagnostic procedures, prognosis determination, and therapeutic efficacy evaluation (3) (**Table 2**).

**Table 2:** The role of lactate in disease.

Disease	Mechanisms	Role of Lactate	References
Cancer	Energy Metabolism, Immune Escape, HIF-1 Stabilization	1. Promotes M2 macrophage polarization, enhancing inflammation and angiogenesis. 2. Upregulates PD-L1 expression, suppressing T-cell functionality. 3. Contributes to acidification of the TME, impairing activation and proliferation of CD8+ and CD4+ T cells. 4. Affects DC maturation and antigen presentation. 5. Histone and Non-histone Lactylation Modifications. 6. Activation of the ERK-STAT3 Signaling Pathway, GPR132, and Notch Pathway. 7. Inhibition of NFAT, NKp46, and mTOR Signaling.	(3, 47–52)
Chronic Inflammation	Inflammatory Response Suppression	1. Reduces inflammation in colitis models via GPR81. 2. Inhibits overactivation of inflammasomes through MCTs in colitis. 3. In murine arthritis models, the expression of the transporter protein SLC5A12 is upregulated, subsequently leading to an enhancement in the production of interleukin-17 (IL-17) by activating the PKM2/STAT3 signaling cascade. 4. Induces immunosuppression in septic acute kidney injury via the PD-1/PD-L1 pathway causing lymphocyte apoptosis.	(53–57)
Cardiovascular Disease	Energy Metabolism, Inflammatory Response and pancreas	Downregulates inflammatory cytokines such as IL-6, IL-8, MCP-1 via GPR81/KLF2 pathways, predicting prognosis and mortality rates.	(58–64)
Respiratory Disease	Energy Metabolism, Inflammatory Regulation	Inhibits IL-33/TGF-β, JNK, ERK, NF-κB pathways, indicating severity and predicting outcomes.	(3, 58–64)
Liver and Pancreatic Diseases	Energy Metabolism, Cell Damage	Suppresses inflammasome activation leading to liveratic damage through GPR81 pathway.	(3, 65)
Brain Injury	Energy Metabolism, Neuroprotection	Used as a biomarker for assessing severity and treating cerebral edema due to its involvement in energy metabolism.	(3)

Deepening the research into lactate metabolism regulatory mechanisms holds promise for developing novel therapeutic strategies. The design and development of small molecule inhibitors targeting key enzymes such as LDH can be employed to regulate lactate production and clearance, thereby reducing tissue damage and organ dysfunction caused by high lactate levels. Through genetic engineering techniques, it is possible to increase the expression of lactate transporters in specific cells, enhancing the body’s capacity for lactate uptake and conversion. The development of CRISPR/Cas9 technology has brought revolutionary progress to the field of gene editing, allowing for precise editing of the genome to alter the expression levels of specific proteins. A study proposed a lactate-responsive gene editing strategy that activates the therapeutic gene editing of signal regulatory protein α (SIRPα) through a lactate-catalyzed chemodynamic approach to reprogram tumor-associated macrophages (TAMs) and improve cancer immunotherapy (66).This indicates that through CRISPR/Cas9 technology, the expression of specific genes, and thus lactate transporters, can be precisely regulated. Additionally, the expression of lactate transporters can also be regulated by controlling metabolic pathways. For example, in yeast, dynamic control of metabolism to produce lactate from glucose substrates can be effectively achieved using a Cre-lox genetic switch. This suggests that by controlling specific metabolic pathways, the production and utilization of lactate, and consequently the expression and function of lactate transporters, can be influenced (67). Moreover, given lactate’s role as an immune signaling molecule, the design of drugs that modulate local lactate concentrations to optimize the immune microenvironment has potential applications. This could lead to enhanced anti-tumor immune responses or suppression of excessive inflammatory reactions. Currently, while there are no drugs specifically designed to regulate lactate levels that have gained widespread clinical application, there are several medications with significant clinical potential. Metformin, a drug widely used in the treatment of type 2 diabetes, indirectly reduces lactate production by inhibiting hepatic gluconeogenesis. Additionally, metformin has anti-inflammatory effects, which may be related to its impact on lactate homeostasis. Studies have shown that metformin can inhibit the activity of nuclear factor κB (NF-κB) through AMP-activated protein kinase (AMPK)-dependent and -independent pathways, thereby exerting anti-inflammatory effects (68). Cells in inflammatory states produce more lactate, so the anti-inflammatory effects of metformin may help maintain normal lactate levels. Sodium bicarbonate also plays an important role in treating lactic acidosis by neutralizing excess lactate and helping to restore blood pH balance. Furthermore, some studies are exploring compounds that can inhibit LDH activity to reduce lactate production. Recent research has shown that nanoenzyme carriers co-loaded with Syrosingopine and lactate oxidase LOD inhibit lactate efflux in tumor cells, achieving highly synergistic therapeutic effects of self-replenishing enhanced catalytic treatment, starvation therapy, and reversal of tumor immune suppression microenvironment (69). In terms of lactate regulation in the tumor microenvironment, while theoretically promising, there are currently no drugs specifically targeting tumor lactate metabolism in routine clinical use. The design and application of drugs that regulate lactate levels is a field full of challenges and opportunities, and future research may develop more specific drugs to improve treatment effects by regulating lactate concentrations in the tumor microenvironment. Measurement of lactate levels is an important tool in the clinical assessment of patient conditions, especially in acute or critically ill patients. An increase in lactate (L) is usually associated with tissue hypoxia and irreversible damage; therefore, measurement of lactate levels is significant for assessing patient prognosis. Arterial blood gas analysis (ABG) is the gold standard for assessing a patient’s oxygenation and ventilation status, particularly suitable for patients in intensive care and emergency situations. (Arterial Blood Gas) Venous blood gas (VBG) testing can be an alternative to ABG in some cases, especially when precise assessment of oxygen partial pressure is not required. Point-of-care testing (POCT) devices provide a rapid means of obtaining clinical information, suitable for situations where rapid adjustments to treatment plans are needed (70). By manipulating these processes, researchers aim to create treatments that fine-tune lactate metabolism for therapeutic benefit.

## 3 Lactic acid and immune evasion

**Figure 2:**
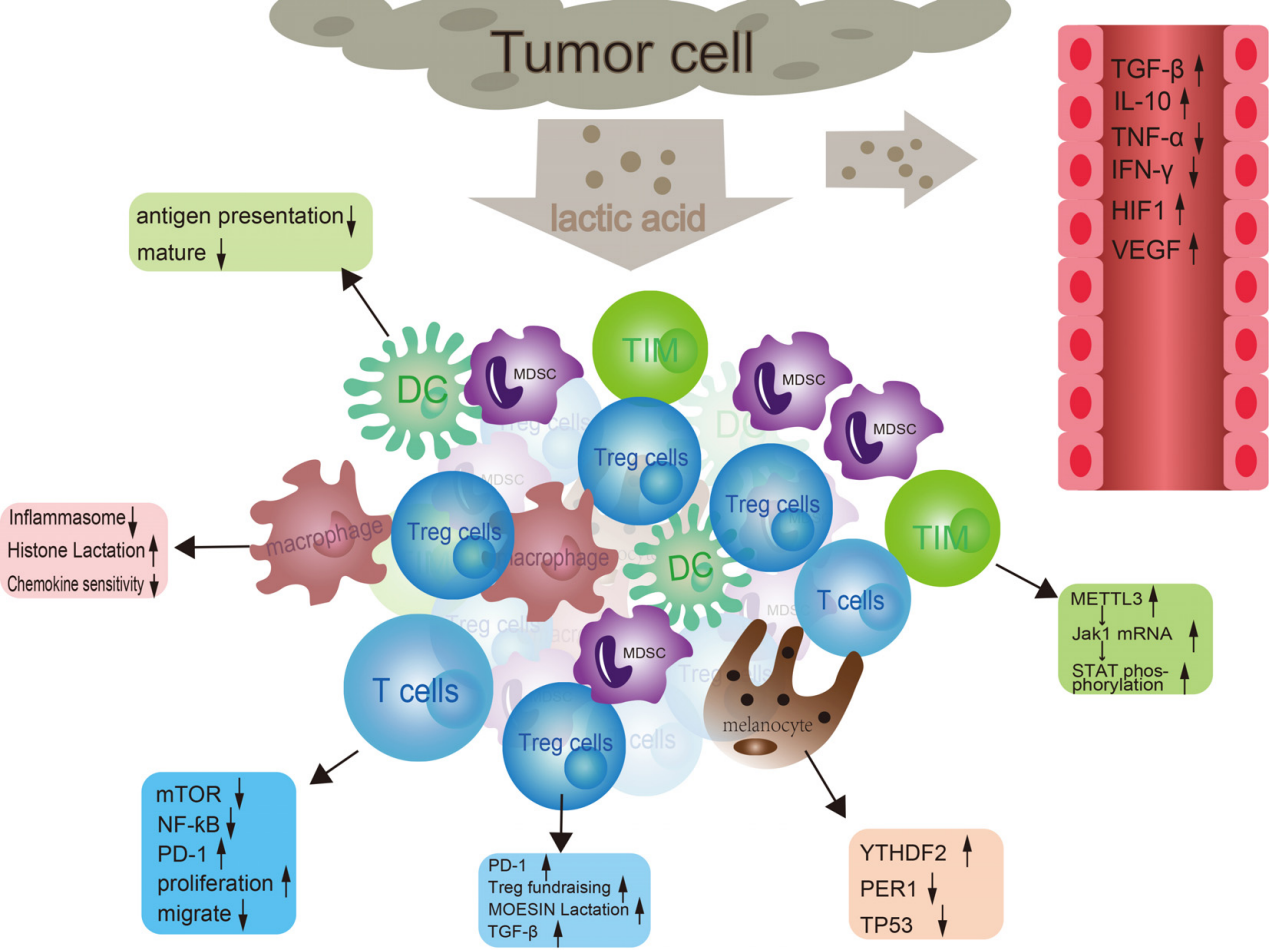
The interaction between tumor cells and immune cells. In the tumor microenvironment, tumor cells secrete lactic acid to affect dendritic cells, macrophages, and regulatory T cells (Tregs). At the same time, tumor cells also regulate T cell function through molecules such as mTOR, NF-ƙB, and PD-1. In addition, tumor cells can also affect the activity of immune cells by releasing signaling molecules such as TGF-β, TNF-α, IFNα, HIF1α, and VEGF. These signaling molecules can activate or inhibit different types of immune cells, thereby affecting tumor growth and metastasis.

Lactic acid regulates tumor immune evasion through multiple mechanisms. (**Figure 2**) High levels of lactic acid can alter the metabolic state and functional polarization of tumor-associated macrophages (71). For instance, lactic acid can regulate the inflammatory response of macrophages by inhibiting the assembly of inflammasomes and the production of cytokines, promoting the transition of macrophages from an M1 to an M2 phenotype, thereby attenuating their ability to phagocytose and kill pathogens (72). Macrophages, under hypoxic conditions, in response to IFN-γ stimulation or bacterial insults, generate lactate through glycolysis, which leads to increased lactylation of histones at promoter regions. This process also promotes the expression of M2-polarization-associated homeostatic genes such as Arg1. Although the regulatory mechanisms underlying histone lactylation remain to be fully elucidated, numerous studies have already demonstrated that histone lactylation plays a significant role in tumor growth (73). Lactic acid concentration can also affect the sensitivity of macrophages to chemokines and their cytoskeletal rearrangement, thereby regulating their ability to migrate to inflammatory sites (74). High levels of lactic acid can lead to a decrease in intracellular pH, which also impacts T cell activation and proliferation. Lactic acid, by influencing cellular metabolic pathways and regulating intracellular signaling pathways such as mTOR and NF-κB, indirectly affects T cell proliferation, differentiation, and effector functions (75). Similarly, lactic acid can impact T cell migration by altering the activity of monocarboxylate transporters on the T cell surface, which in turn adjusts the intracellular metabolic state and interactions with the extracellular matrix (76).

In the field of oncology, immune evasion refers to how cancer cells employ a variety of mechanisms to bypass the body’s inherent immune defense system, rendering the immune system unable to effectively recognize and eliminate them. This allows cancer cells to survive, proliferate, and disseminate within the body. Regulatory T cells (Tregs) are a key mechanism by which tumors evade the immune response. Cancer cells can induce the accumulation of immunosuppressive cells such as Tregs and myeloid-derived suppressor cells (MDSCs), which inhibit the function of other immune cells, or they may secrete soluble cytokines that interfere with the activity of immune cells (77). Cancer cells may also downregulate the expression of antigens on their surface that are normally recognized by immune cells, rendering them unidentifiable; concurrently, they can express immune checkpoint molecules like PD-L1, which upon binding to PD-1 on T cells, inhibit T cell activation, leading to immune tolerance against cancer cells (78). Cancer cells can produce hypoxia-inducible factors, inflammatory cytokines, and induce the formation of new blood vessels to remodel the tumor microenvironment in a way that is detrimental to immune cell infiltration (79, 80). Cancer cells, with their high mutation rates, can give rise to novel antigenic epitopes, such that even if there is an immune response against the original antigens, it may not effectively counteract the ever-changing cancer cells. These cancer cells undergo selection by the immune system and eventually evolve into clones capable of evading immune clearance. Through the aforementioned mechanisms and other intricate interactions, cancer cells successfully achieve immune evasion, allowing them to persist within the host organism.

The accumulation of lactate in the tumor microenvironment is a significant factor contributing to immune evasion in tumors. In highly glycolytic tumor microenvironments, Treg cells uptake lactate via MCT1 and overexpress PD-1, which can lead to the failure of PD-1 blockade therapy (81). Lactate, acting as a critical metabolic signaling molecule, enhances the immunosuppressive environment for tumors by regulating MOESIN lactylation in Treg cells, thereby bolstering TGF-β signaling pathways. This discovery holds significant implications for understanding the mechanisms of tumor immune evasion and for the development of novel immunotherapeutic strategies (82). Research has shown that lactate accumulated in the tumor microenvironment can increase the expression of m^6^A methyltransferase METTL3 in tumor-infiltrating myeloid cells (TIMs) through H3K18 lactylation. In TIMs, METTL3-mediated m6A modification of Jak1 mRNA enhances the translation efficiency of Jak1 protein and subsequent STAT3 protein phosphorylation process, thereby strengthening the immunosuppressive function of TIMs, which is associated with poorer prognosis and tumor progression in colorectal cancer patients (83). Further research demonstrates that histone lactylation facilitates the expression of the YTHDF2 protein, which is an m^6^A modification reader protein. YTHDF2 recognizes and degrades the mRNA of two tumor suppressor genes, PER1 and TP53, thereby accelerating the formation and progression of melanoma (84). Inhibiting histone lactylation can effectively suppress the proliferation, migration, and clonogenic capacity of melanoma cells, thereby slowing down tumor progression.

Tumor cells, due to their unique metabolic characteristics, produce a large amount of lactate. An increase in lactate concentration within the tumor tissue leads to a decrease in local pH values, creating an acidic microenvironment. This acidic milieu not only favors the proliferation and invasion of tumor cells but also can suppress the function of immune cells (85). Lactic acid can directly inhibit the activity of immune cells, such as by suppressing the proliferation and cytotoxic functions of T cells. It does so through inhibiting the expression of cell cycle-related proteins and the NF-κB signaling pathway, thereby reducing the anti-tumor effects of T cells (86). Moreover, a high lactate environment can also impair the maturation and antigen-presenting function of dendritic cells, thereby weakening their ability to initiate and sustain anti-tumor immune responses (87). In the tumor microenvironment, high levels of lactate can influence the cytokine network, leading to the induction or suppression of certain immune regulatory factors. For example, lactate can upregulate the expression of immunosuppressive cytokines such as TGF-β and IL-10, while concurrently downregulating pro-inflammatory cytokines like IFN-γ and TNF-α, thus further contributing to the establishment of an immunosuppressive state (88). Lactic acid promotes the production of Vascular Endothelial Growth Factor (VEGF), which induces the formation of new blood vessels in tumors. These newly formed vessels tend to be leaky, making it difficult for immune cells to effectively reach the core of the tumor. Concurrently, lactic acid may also influence lymphatic vessel generation, reducing the drainage efficiency of immune cells, thereby enabling tumors to more readily evade surveillance and attack by the immune system (23, 73). The accumulation of lactic acid within the tumor microenvironment facilitates immune evasion by tumors through multiple pathways, allowing cancer cells to evade recognition and elimination by the immune system, thereby enabling their continuous growth and spread. Most research generally agrees that lactic acid exerts immunosuppressive effects within the tumor microenvironment; however, recent studies have demonstrated a contrasting effect, showing that increasing the stemness of CD8+ T cells can suppress tumor growth with a positive immunoenhancing outcome (89). Surprisingly, lactate also regulates immune escape by paracrine activation of GPR81 on the surface of stromal dendritic cells (73, 90).

Recent studies have found that regulatory T cells (Tregs) play a key role in tumor immune evasion, with lactate playing an essential part in this process. Research indicates that the uptake of lactate by Tregs is crucial for the effectiveness of CTLA-4 blockade therapy. Lactate promotes RNA splicing mediated by USP39, enhancing the expression of CTLA-4 in Tregs in a Foxp3-dependent manner. Furthermore, the absence of USP39 leads to the loss of Tregs function and can trigger fatal autoimmune reactions, highlighting the importance of USP39 and lactate in regulating Tregs function and antitumor immunity (91). Additional research has found that the m6A RNA demethylase ALKBH5 affects the accumulation of Tregs and MDSCs in the tumor microenvironment by regulating the expression of Mct4/Slc16a3 and lactate content, thereby influencing the response to anti-PD-1 therapy (92). This suggests that ALKBH5 may be a potential target for regulating the tumor microenvironment and enhancing the effectiveness of immunotherapy (92). The impact of lactate on Th17 cells is also a focus of research. Studies have found that lactate can significantly reduce the ability of Th17 cells to produce IL-17A, while increasing Foxp3 expression through ROS-driven IL-2 secretion, promoting the transformation of Th17 cells into Tregs, which may play a role in suppressing Th17 cell-driven inflammation and autoimmune reactions (93). Additionally, lactate directly impairs the cytotoxic ability of T cells and natural killer (NK) cells (94, 95). It limits the activity of glycolytic enzymes in T cells, reducing the synthesis of pro-inflammatory cytokines and promoting the shift of T cells to a tolerant state (94). In NK cells, the presence of lactate reduces the expression of the activating receptor NKp46 and the production of perforin and granzyme B, which are essential for NK cell cytotoxicity (94). Lactate also inhibits the production of IFN-γ by affecting the NFAT signaling pathway, which plays a key role in the activation of T cells, NK cells, and other immune cells (94). Therefore, the activity of lactate dehydrogenase A (LDHA) is crucial for the antiviral and antitumor functions of NK cells (95). In the tumor microenvironment, high concentrations of lactate have been found to inhibit the antigen-presenting function of dendritic cells (DCs), reduce the activity of CD8+ T cells, render them anergic and apoptotic, thereby impairing cytotoxic activity and cytokine secretion (96–98). In summary, the role of lactate in the tumor microenvironment is far more complex than we previously recognized. It not only directly inhibits the function of immune cells but also promotes the accumulation of immunosuppressive cells and suppresses the function of immune-activating cells through various metabolic pathways and molecular mechanisms, playing a central role in tumor immune evasion. These findings provide us with new perspectives for developing immunotherapy strategies targeting lactate metabolism and signaling pathways, in hopes of overcoming tumor immune evasion and improving the success rate of cancer treatment.

## 4 Targeting lactate metabolism to improve immune escape

Targeting lactate metabolic pathways and blocking lactate transporters such as MCT1 and MCT4 can restrict tumor cell efflux of lactate and inhibit glycolysis, potentially conferring anti-tumor effects (99). Through pharmacological or genetic engineering means to increase lactate metabolic enzymes, such as lactate dehydrogenase (LDH), can accelerate the clearance of lactate, thereby improving local acidic environments and enhancing the activity of immune cells. For instance, by inhibiting LDHA (lactate dehydrogenase A subunit) to reduce lactate production, it can help reverse the acidification of the tumor microenvironment and boost T cell activity (81, 95, 100). Research indicates that modulating the lactate metabolic pathways within immune cells can enhance their anti-tumor activity. For instance, by increasing immune cells’ utilization of lactate rather than perceiving it as a toxic byproduct, it can serve as an adaptive mechanism to improve the functionality of immune cells in hypoxic environments (81, 101). Recent research has revealed that lithium carbonate interferes with the ATPase proton pump on the lysosomal membrane, preventing cytoplasmic hydrogen ions from entering the lysosome. This indirect action promotes the redistribution of the DG-PKC θ signaling complex on the lysosomal membrane to the mitochondrial membrane, enhancing mitochondria’s uptake and utilization of lactate, thus bolstering its anti-tumor effects (102). Moreover, lactate oxidase encapsulated in nanocapsules also enhances the anti-tumor immune therapeutic effects of T cells (103). Co-delivery systems are created using liposomes that encapsulate quercetin and doxorubicin, with a legumain-specific responsive hairpin peptide KC26 conjugated to the liposome surface, forming dual-targeting KC26-Lipo liposomes. The KC26-Lipo system works by inhibiting lactate metabolism in breast cancer, repolarizing tumor-associated macrophages (TAMs), and suppressing tumor angiogenesis through the HIF-1α/VEGF pathway. Quercetin is an inhibitor of monocarboxylate transporters (MCTs) and effectively reduces the production and secretion of lactate by tumor cells (79).

However, several significant challenges remain. For instance, when intervening in lactate metabolism, it is essential to ensure that the approach effectively kills tumor cells without impairing the functionality of immune cells. The design of drugs with both adequate therapeutic efficacy and safety is needed, capable of precisely targeting enzymes or transporters involved in the lactate metabolic pathway within the body. Lactic acid’s influence on the anti-tumor efficacy of immune checkpoint inhibitors underscores the desirability of targeting tumor metabolism while simultaneously enhancing the effectiveness of these inhibitors. The tumor microenvironment is highly complex, with lactic acid playing roles in multiple signaling pathways and involving various cell types, necessitating a comprehensive understanding and precise regulation. Since different patients’ tumors may exhibit distinct metabolic characteristics, this implies that strategies for intervening in lactate metabolism need to be personalized. In essence, addressing the impact of lactic acid on the therapeutic outcome of immune checkpoint inhibitors requires a holistic comprehension and tailored approaches in light of the heterogeneous nature of tumor metabolism across patients.

Targeting lactate metabolism as an emerging cancer treatment strategy is gradually being proven by research. However, this therapeutic approach also comes with potential risks and side effects that require careful consideration in clinical application. First, the role of lactate in the tumor microenvironment is a double-edged sword. It is not only a source of energy for tumor cells but also a carbon source for immune cells, contributing to enhanced immunity. Studies indicate that the impact of lactate on immune strategies in lymphoma suggests that targeting lactate metabolism may disrupt normal immune functions, leading to immune evasion or suppression. Therefore, we need to deeply understand the complex role of lactate in immune responses and precisely regulate its metabolic pathways to avoid damaging the body’s immune defense capabilities. Additionally, treatments targeting lactate metabolism may cause a range of immune-related adverse events (irAEs), including skin reactions, endocrine dysfunction, hypertension, diarrhea, liver issues, and ocular toxicity (104–106). These adverse events may be related to the drug’s direct action on the target or to immune reactions caused by the drug. Therefore, for patients receiving such treatments, their health status needs to be closely monitored, and appropriate management strategies should be prepared to mitigate or prevent the occurrence of these adverse events. Targeting lactate dehydrogenase (LDH) may lead to cell cycle dysregulation and increased sensitivity to DNA damage response, affecting the survival of tumor cells (107). However, this treatment strategy may also have adverse effects on normal cells, as LDH is expressed in various cell types, and its inhibition may disrupt normal cell functions. This requires us to pursue higher selectivity in drug design to reduce adverse effects on normal cells. Treatments targeting lactate metabolism may also induce inflammatory toxicity involving any organ system (108). Although most of these inflammatory toxicities can be resolved through systemic immunosuppression, fatal toxicity events can still occur, and interruptions and discontinuations of immunotherapy due to toxicity are also relatively common. Therefore, for such treatments, we need to be well-prepared, including early recognition of inflammatory toxicity, timely intervention, and effective management. In summary, targeting lactate metabolism has tremendous potential in cancer treatment but also comes with potential risks and side effects on normal cells and overall immune function. In developing and applying such treatment strategies, we need to carefully weigh the pros and cons and take appropriate management measures to mitigate the impact of adverse events. Through precise target selection, optimized drug design, and strict patient management, we can maximize the therapeutic efficacy of targeting lactate metabolism while minimizing its adverse effects on the body.

The role and importance of glutamine metabolism in cancer cells have been extensively studied and recognized. Glutamine is not only an important carbon and nitrogen source for the growth and proliferation of cancer cells but also plays a key role in maintaining redox balance, providing NADPH, and supporting the synthesis of lipids, proteins, and nucleic acids (109). Furthermore, glutamine metabolism is closely related to the survival, proliferation, metastasis, and invasive ability of cancer cells (110). Fatty acid oxidation (FAO) is another pathway for cancer cells to meet energy demands when glucose supply is limited. Inhibition of key metabolic enzymes may provide new strategies for cancer treatment. One-carbon metabolism is crucial for DNA synthesis, repair, and cellular methylation status in cancer cells. This pathway supports the synthesis of proteins, nucleic acids, and lipids by providing one-carbon units, while maintaining cellular redox balance (111). Inhibition of key enzymes in the one-carbon metabolic pathway may disrupt the proliferation and epigenetic regulation of cancer cells. The pentose phosphate pathway (PPP) not only provides NADPH to maintain redox balance but also provides pentoses for nucleic acid synthesis. Enhancement or inhibition of the PPP may affect the antioxidant capacity and DNA synthesis of cancer cells, thereby affecting their survival and proliferation. Autophagy is an important mechanism for cancer cells to maintain survival during nutrient deprivation. Activation or inhibition of autophagy may affect the resistance of cancer cells to immune attacks, providing new strategies for immunotherapy (112). Glutamate metabolism also plays a significant role in cancer cells. Glutamate is not only a neurotransmitter but also a precursor of glutathione, which is essential for antioxidant stress and maintaining cellular homeostasis. In summary, glutamine metabolism and its related pathways play a key role in the survival, proliferation, and resistance to treatment of cancer cells. Exploring the key enzymes and regulatory mechanisms of these metabolic pathways will provide important targets for the development of new cancer treatment strategies.

## 5 Discussion

The relationship between lactate metabolism and immune escape manifests across multiple layers, forming a complex interplay that plays a pivotal role in the development and progression of tumors as well as their treatment. Tumor cells, due to aberrant glycolysis (Warburg effect), produce large quantities of lactate, which results in acidification of the TME. This acidity suppresses the activity of immune cells, thereby assisting tumor cells in evading immune surveillance (113). High levels of lactate can also induce the expression of certain immune suppressive cells and factors, such as Tregs, MDSCs, TGF-β, IL-10, VEGF, among others, further promoting an immunosuppressive state. The presence of lactate alters the local energy supply situation, potentially depriving immune cells of sufficient nutrients to maintain their normal functions. Lactic acid can directly or indirectly impact intracellular signaling pathways within immune cells, regulating their proliferation, differentiation, and functionality, thereby affecting their capacity to combat tumors (95). Lactate can also enhance anti-tumor immunity by increasing the stemness of CD8+ T cells (89).

Tumor cells, through the overexpression of lactate dehydrogenase A (LDHA) among other mechanisms, lead to excessive lactate production. The accumulation of lactate not only lowers the local pH, creating an acidic tumor microenvironment that suppresses the function of immune effector cells such as macrophages and natural killer cells, but also indirectly promotes immune evasion by influencing the expression of cell surface receptors and signaling pathways. Developing inhibitors targeting key enzymes in lactate metabolism, such as LDHA, can reduce lactate production by tumor cells, thereby improving the tumor microenvironment and enhancing the infiltration and killing ability of immune cells against tumors. Concurrently, research into inhibitors that target monocarboxylate transporters (MCTs), which facilitate the efflux of lactate out of tumor cells, can be pursued to block lactate export, further exacerbating intratumoral acidosis, and ultimately driving tumor cell death. In the future, there is a pressing need to proactively explore several avenues: developing therapeutic strategies to enhance immune cell tolerance to lactate, such as upregulating the expression of MCT1 or MCT4, thereby improving the transport and utilization efficiency of lactate within immune cells, ensuring they can maintain normal proliferation, differentiation, and anti-tumor functions even within an acidic environment. Based on gene expression profiling and metabolomics analysis of patient-derived tumor tissues, it is crucial to identify personalized lactate metabolic targets and integrate these findings into the formulation of individualized immunotherapy plans. This could involve the concurrent use of lactate metabolism modulators, like MCT1 inhibitors or LDHA inhibitors, along with PD1/PD-L1 antibodies and CAR-T cell therapies. Furthermore, conducting preclinical trials is necessary to deeply investigate the synergistic mechanisms between lactate metabolism regulators and immunotherapies, aiming to uncover the most efficacious dosage combinations and drug administration regimens that optimize treatment outcomes while minimizing side effects. Lastly, utilizing lactate and its metabolic byproducts as biomarkers would allow for real-time monitoring of the dynamic changes in the tumor microenvironment, enabling the assessment of treatment response, predicting patient prognosis, and furnishing a basis for informed clinical decision-making.

Lactate metabolism modulators, particularly lactate dehydrogenase A (LDH-A) inhibitors, show great potential in cancer treatment. These modulators enhance the function of immune cells by regulating lactate levels in the tumor microenvironment, thereby improving the effectiveness of immunotherapy. For instance, the LDH-A inhibitor FX-11 (BGB-A1217) is being evaluated in clinical trials for its safety and efficacy in patients with solid tumors, with the expectation that it will enhance the activity of immune cells and increase the response rate to immunotherapy by reducing lactate levels in the tumor microenvironment (114). When designing clinical trials, factors such as the dose of lactate metabolism modulators, administration methods, duration of treatment, and combination with other treatments should be considered. LDH-A not only serves as a biomarker for tumor diagnosis and prognosis but also represents an ideal target for tumor treatment. Additionally, metformin, as an AMPK activator that affects lactate metabolism, is currently being assessed for its effectiveness in combination with PD-1 inhibitors in treating non-small cell lung cancer (92). In the context of personalized medicine, individualized treatment plans can be developed based on the variations in lactate levels and the expression of lactate metabolism-related genes in the tumor microenvironment. By evaluating the lactate metabolism characteristics of patients, a patient population most likely to benefit from treatment with lactate metabolism modulators can be selected. There is a significant negative correlation between lactate metabolism-related characteristics and antitumor immunity and immune cell infiltration imbalance, and a positive correlation with pro-tumor signal transduction. Using this characteristic, machine learning models have predicted immunotherapy response and prognosis with outstanding predictive performance. In terms of biomarker development, the discovery and validation of lactate metabolism-related biomarkers can predict patient response to treatment and can be used for patient selection, treatment response monitoring, and prognosis assessment. In summary, the role of lactate in the tumor microenvironment has profound implications for the treatment and prognosis of clinical cancer patients. Intervention strategies targeting lactate metabolism may provide new avenues for cancer treatment. Future research should further explore the combination of lactate metabolism modulators with other immunotherapy strategies to improve treatment outcomes.

Currently, in the field of cancer treatment research, intervention strategies targeting lactate metabolism constitute a significant area of investigation. Lactate plays multiple roles within the tumor microenvironment, not only promoting proliferation of tumor cells themselves but also influencing immune cell function by regulating microenvironmental acidity, activating specific receptors and signaling pathways, thereby suppressing the body’s immune response to tumors and enhancing tumor resistance. Therefore, designing drugs that precisely target enzymes or transport proteins involved in lactate metabolism represents one of the key challenges in achieving effective anti-tumor therapies. Simultaneously, to ensure that such drugs effectively suppress tumor growth without compromising the normal functioning of the immune system, a deep understanding of the mechanisms by which lactate operates within various signaling pathways and its complex impacts on multiple cell types is required. Furthermore, the integration of therapies targeting tumor metabolism with immune checkpoint inhibitors holds promise for realizing better clinical treatment outcomes.

In the field of cancer treatment, enhancing antitumor immune responses through precise metabolic modulation is gradually becoming a promising research direction. The diversity and uniqueness of these strategies offer broad application potential for clinical treatment. Firstly, enhancing oxidative stress levels in the tumor microenvironment, by increasing the concentration of reactive oxygen species (ROS), not only promotes DNA damage and death in tumor cells, enhancing immunogenicity, but also aids in the maturation of dendritic cells (DCs), thereby more effectively presenting tumor antigens and activating T cells. Inhibiting the activity of key enzymes such as lactate dehydrogenase (LDH) improves the effectiveness of immunotherapy, opening new avenues to overcome tumor immune evasion (115). Secondly, modulating glutamine metabolism provides a new strategy for tumor treatment. Glutamine, as an important energy and biosynthetic precursor for tumor cells, has its metabolism inhibited to restrict tumor cell growth. By inhibiting key enzymes such as glutaminase (GLS), not only is tumor cell biosynthesis blocked, but T cell function may also be enhanced, offering a new perspective for tumor immunotherapy (116, 117). Additionally, targeting fatty acid metabolism is an important strategy to enhance antitumor immune responses. Many tumor cells rely on fatty acid synthesis pathways to support their rapid proliferation. Inhibiting key enzymes such as fatty acid synthase (FASN) or acetyl-CoA carboxylase (ACLY) can reduce the energy supply of tumor cells, while potentially increasing immune cell attacks on tumors, providing new strategies for cancer treatment (118, 119). Activating metabolic pathways in immune cells is also crucial. By promoting glycolysis and oxidative phosphorylation (OXPHOS) in T cells and NK cells, their antitumor activity can be enhanced. Using costimulatory molecules such as CD28 or cytokines like IL-2 can enhance the metabolic adaptability and antitumor functions of T cells, providing new ideas for improving the effectiveness of immunotherapy (120–122). Lastly, modulating amino acid metabolism, especially tryptophan metabolism, is equally important for inhibiting tumor growth and enhancing immune responses. Amino acids play a key role in multiple steps of T cell antitumor immunity, including activation, proliferation, effector function, memory formation, and functional exhaustion, providing a new direction for cancer treatment (123). In summary, multiple metabolic modulation strategies can effectively enhance antitumor immune responses. These strategies include enhancing oxidative stress, modulating glutamine metabolism, targeting fatty acid metabolism, activating immune cell metabolic pathways, and modulating amino acid metabolism. Each strategy has its unique mechanism of action and significant clinical application potential, collectively pointing to a new direction for the future of cancer treatment.

In the field of cancer treatment, the development and application of lactate metabolism modulators are becoming a research hotspot. Particularly, small molecule inhibitors targeting lactate dehydrogenase A (LDH-A) can alter the tumor microenvironment, enhance the infiltration and function of immune cells, and provide new strategies for cancer treatment. Pyruvate dehydrogenase (PDH) is a key enzyme that converts pyruvate to acetyl-CoA. The development of PDH activators that stimulate PDH could promote the oxidative phosphorylation of pyruvate and reduce lactate production. This not only helps regulate the metabolic state of tumor cells but also enhances the function of immune cells. It is also crucial to study the metabolic characteristics and immune regulatory effects of lactate in different types of cancer. The immunosuppressive effects of lactate may vary in different cancers. In melanoma, the accumulation of lactate can suppress the function of cytotoxic T cells, reduce the production of cytokines, and thus weaken the immune system’s attack on tumors (20). In contrast, in triple-negative breast cancer, lactate promotes tumor growth and invasion by affecting the differentiation and function of dendritic cells (124). Future research may reveal the specific roles of lactate in different cancers, providing new targets for the development of targeted treatment strategies. Additionally, studying the crosstalk between lactate metabolism and other metabolic pathways, such as fatty acid metabolism and amino acid metabolism, may reveal new synergistic mechanisms, offering new strategies for cancer treatment. Utilizing advanced imaging technologies and single-cell metabolic analysis to study the spatiotemporal changes in lactate metabolism during tumor development and how these changes affect the behavior and function of immune cells will help to more precisely understand the dynamic role of lactate metabolism in the tumor microenvironment. Finally, studying the combination of lactate metabolism modulators with other immunotherapy strategies, such as immune checkpoint inhibitors and CAR-T cell therapy, to enhance efficacy is essential. This combination therapy strategy may enhance the immune response by modulating lactate metabolism, providing more effective treatment options for cancer patients.

In summary, these research directions will help us to more comprehensively understand the role of lactate in tumor immune evasion and provide a scientific basis for the development of new immunotherapy strategies. Through these studies, we hope to improve the effectiveness of cancer treatment, especially for patients who do not respond well to traditional therapies.
